# Mississippi Valley Association of Dental Surgeons

**Published:** 1845-11

**Authors:** 


### MISSISSIPPI VALLEY ASSOCIATION OF DENTAL SURGEONS.

*First Annual Meeting.*

In our last, we had the pleasure of announcing the establishment in our sister city, Cincinnati, of a College for instruction in the profession of Dentistry. Since that time, we have received a small pamphlet under the above title, from which it appears, that on the 19th of August last, there was, in the same city, a meeting of Surgeon Dentists, for the purpose of elevating the character of their profession. About a dozen gentlemen were in attendance. The officers were Joseph Taylor, President, and W. B. Ross, Secretary. An address was read from Dr. Brown of St. Louis, on Artificial Palates and Obturators; another by Dr. Rogers of Cincinnati, on Caries in Deciduous Teeth, and another from the president, Dr. Taylor, on the use of Mercurial Remedies as medicinal agents, and their influence on the dental organs. These are interesting topics, and we hope the future meetings will bring forth many other papers on subjects of equal importance. We regret to observe the absence from this meeting, of a number of distinguished dentists, resident in this and other cities of the West. We trust they will contribute by their presence and their pens to the exercises of the future meetings of the association; and thus assist in promoting the improvement and dignity of a profession, which has hitherto had to carry a heavy weight of empiricism: a simultaneous effort should be made to throw it off. D.

